# NK Cell Autoreactivity and Autoimmune Diseases

**DOI:** 10.3389/fimmu.2014.00027

**Published:** 2014-02-04

**Authors:** Alessandro Poggi, Maria Raffaella Zocchi

**Affiliations:** ^1^Molecular Oncology and Angiogenesis Unit, IRCCS AOU San Martino-IST, Genoa, Italy; ^2^Division of Immunology, Transplants and Infectious Diseases, Scientific Institute San Raffaele, Milan, Italy

**Keywords:** NK cells, autoreactivity, autoimmunity, NKG2D, DNAM1, regulatory NK cells, mesenchymal stromal cells, LAIR1

## Abstract

Increasing evidences have pointed out the relevance of natural killer (NK) cells in organ-specific and systemic autoimmune diseases. NK cells bear a plethora of activating and inhibiting receptors that can play a role in regulating reactivity with autologous cells. The activating receptors recognize natural ligands up-regulated on virus-infected or stressed or neoplastic cells. Of note, several autoimmune diseases are thought to be linked to viral infections as one of the first event in inducing autoimmunity. Also, it is conceivable that autoimmunity can be triggered when a dysregulation of innate immunity occurs, activating T and B lymphocytes to react with self-components. This would imply that NK cells can play a regulatory role during adaptive immunity; indeed, innate lymphoid cells (ILCs), comprising the classical CD56^+^ NK cells, have a role in maintaining or alternating tissue homeostasis secreting protective and/or pro-inflammatory cytokines. In addition, NK cells display activating receptors involved in natural cytotoxicity and the activating isoforms of receptors for HLA class I that can interact with healthy host cells and induce damage without any evidence of viral infection or neoplastic-induced alteration. In this context, the interrelationship among ILC, extracellular-matrix components, and mesenchymal stromal cells can be considered a key point for the control of homeostasis. Herein, we summarize evidences for a role of NK cells in autoimmune diseases and will give a point of view of the interplay between NK cells and self-cells in triggering autoimmunity.

## Introduction

Natural killer (NK) cells are one of the main components of innate immunity [reviewed in Ref. (1–7)]. It is thought that they provide the body with a strong defense against microorganisms, such as viruses and bacteria, together with their efficient action in limiting neoplastic cell growth (1). The functional definition of NK cells, that is their ability of killing other cells without any prior stimulation, implies that different cell populations can have the functional characteristics of NK cells without sharing a common phenotype. The large majority of the surface markers able to identify this cell population are actually expressed by other kinds of lymphocytes leading to an intrinsic difficulty in defining a cell as an NK cells on the basis of phenotype. As several other components of the innate arm of the immune system, NK cells can secrete cytokines and chemokines. Both activation of cytolytic machinery and secretion of regulating soluble factors are dependent on a wide number of surface and intracellular receptors that, interacting with the appropriate ligand, can lead to activation or inhibition of a given cell function. As always in a biological system, the balance between these opposite signals is responsible for the final outcome in the microenvironment; thus, NK cells can influence and regulate the activities of adaptive immune responses, including T cells [reviewed in Ref. (8)] and dendritic cells (DCs) (9, 10) through well identified surface receptors. Recent findings have pointed out that NK cells may play important roles in autoimmune disorders; indeed, a genetic correlation between NK cell expression of HLA-I receptors and autoimmune diseases has been shown. In addition, it appears that NK cells may play opposite roles with both regulatory and inducer activity in some autoimmune diseases (11–25).

## Functional Balance between Activating and Inhibiting Signals in NK Cells

It is well known that the functional behavior of NK cells can be regulated by positive and negative signals. A detailed analysis of positive and negative NK cell receptors is reported elsewhere (6). Roughly, two main systems of molecular regulators are expressed on NK cells: the first one is represented by invariant NK cell receptors for HLA-I while the second one is composed of several receptors which do not bind HLA-I. The molecular and functional characteristics of NK cell receptors for HLA-I have been extensively analyzed (26–28): briefly, killer immunoglobulin-like inhibitory receptors (KIRs) and C-lectin-type-inhibitory receptors (CLIRs) can recognize either unique or several HLA-I alleles blocking NK cell function. Some members of these receptors can be expressed on NK cells also in an activating isoform that, in the extracellular portion, is apparently identical to the inhibiting one, indicating that the same HLA-I allele product can be positively recognized as well. Furthermore, in some instances, only the activating form of a member of KIR family has been identified, although it is not still defined unequivocally its corresponding HLA-I ligand. All these findings would render the scenario of NK cell receptors for HLA-I much more complicated than it was supposed in the late 90s (29, 30). Regarding the non-HLA-I receptors present on NK cells, some are of the activating type such as CD69, NKp30, NKp44, NKp46, NKG2D, and DNAM1 (31–33), while others are of the inhibiting type as LAIR1 (34). It should be noted that the peculiar behavior of the 2B4 receptor, which can deliver an activating signal when the signal transducer called SAP/SH2D1A is present in the cytoplasm; but in some instances it can deliver an inhibiting signal also in the presence of this transducer [reviewed in Ref. (35, 36)]. It is commonly thought that NK cells do not aggress self-cells because the balance between negative and positive signals is always in favor of the negative regulation: this balance is broken when self-cells do not express HLA-I (as during viral infections) or up-regulate natural ligands for activating receptors as it happens during tumor transformation (10, 37).

## Evidence for the Recognition of Self-Cell by NK Cells

Like T lymphocytes, NK cells should not recognize autologous cells, unless autoreactivity is triggered, potentially leading to an autoimmune disease. Based on the original definition of NK cells (1), in principle a self-cell can be killed by NK cells without any previous stimulation. To avoid this damage, a self-cell is equipped with two major molecular mechanisms: (a) strong expression of HLA-I antigens able to deliver inhibiting signals to NK cells; (b) low levels or lack of expression of surface ligands essential for triggering NK cell activation [reviewed in Ref. (8, 37)]. In the latter context, also the down-regulation of ligands for co-receptors of NK cell activation can play a key role in avoiding self-aggression [reviewed in Ref. (4, 38)].

It is becoming evident that NK cells can recognize self-cells, which express ligands for activating receptors (8); indeed, NK cells can aggress both T and antigen presenting cells (APCs) upon triggering with toll-like receptor (TLR) or stimulation with IL2 or IL15 cytokines. These stimuli lead to the up-regulation of NKG2D receptor or to the neo-expression of CD69 and NKp44, which in turn can trigger cytolytic activity and cytokine production (31, 39). On the other hand, several stimuli conceivably acting through the T cell receptor/CD3 complex, such as phytohemoagglutinin (PHA), alloantigens, superantigens, and antigenic peptides, can induce the neo-expression of NKG2D ligands (NKG2DL) on CD4^+^ and CD8^+^ T lymphocytes [reviewed in Ref. (8)]. Moreover, also microorganisms as HIV or *Mycobacterium tuberculosis* can trigger NKG2DL expression on CD4^+^ T cells and T regulatory (Treg) cells (40, 41). The NKG2DL are represented by stress-induced MHC class I-related molecules, such as MICA/B, or the UL16 binding proteins (ULBPs), that are indeed recognized not only by NK cells but also by a large number of “unconventional” T lymphocytes, as γδ T and NKT cells (11, 12, 42–44). It is conceivable that even CD8^+^ memory T cells could be triggered through NKG2DL; all these cell populations can lead, acting alone or together, to autoreactivity (11). Indeed, the duty of innate immunity is to clear the body from a specific pathogen or impede the development of cancer; thus, one can consider autoimmunity as a drawback of a defective lymphoid stress surveillance that does not limit properly the dissemination of infected or malignant cells and does not maintain tissue integrity, leading to an altered adaptive immune response. In addition, also the poliovirus receptor (PVR) or nectin-2, both ligands for DNAM1 (45) can be expressed on activated or HIV-infected CD4^+^ T cells possibly leading to NK cell recognition through the DNAM1 activating receptor. To our knowledge, no reports are present so far in the literature on the possible interactions between activated T cells and NK cell receptors, such as natural cytotoxicity receptors and/or 2B4, although the 2B4 ligand CD48 can be expressed on T, B, and NK cells [reviewed in Ref. (46)]. It has been shown in a mouse model that blocking of 2B4 with a 2B4-fusion protein inhibits the generation of autoimmune hepatitis (AIH) suggesting that a still undefined 2B4^+^ lymphocyte subset can be involved (47). This deserves further studies in humans to better clarify the molecular mechanisms of NK cell-T lymphocyte cross-talk. Nevertheless, these findings strongly indicate that NK cells can strikingly regulate T cell responses influencing adaptive immunity. In the adaptive immune response, APCs take a key role; indeed, APC can adequately expose the peptide antigen to allow its recognition by T cells (48). Different kinds of APC, with a reported different capacity of presenting the peptide antigen, can be identified (49–51). Focusing our analysis on monocyte and monocyte-derived dendritic cells (moDCs), it is known that NK cells can actively interact with these APC that produce interleukin 12 (also known as NK stimulating factor), which triggers both proliferation and cytolytic activity of NK cells (52). In turn, NK cells can produce cytokines, as TNFα, which contribute to DC cell maturation. Several reports have shown that IL2-activated NK cells can lyse self-APC and that NK–APC interaction may lead to cytokine production (9, 10, 49, 53, 54). Importantly, this interaction can be mediated by different activating receptors, including some natural cytotoxicity receptors, and by NKG2D or DNAM1 (9, 54–59). In addition, ligands for NKG2D can be up-regulated on APC upon stimulation with TLR-ligands, further supporting the idea that microbial infections can evoke an autoreactive response that leads to a limited adaptive immune response. Indeed, the NK cell-mediated elimination of a given APC before antigen presentation to T cells should conceivably impede an optimal T cell activation [reviewed in Ref. (10, 49)]; thus, also the second player of the adaptive immune response can be shut down by NK cells. Finally, on epithelial and mesodermal-derived cells, as well as on leukocytes, adhesive ligands such as the intercellular adhesion molecule-1 (ICAM1) can be up-regulated upon triggering by TLR or inflammatory cytokines, including IFNγ and TNFα (60). The counter receptor of ICAM1 is the lymphocyte function associated antigen-1 (LFA1), which is a major player of leukocyte-to-cell adhesion and NK cell activation [reviewed in Ref. (60–64)]. Of course, stress signals can up-regulate the ligands for NK cell activating receptors also on this cell population, favoring the NK cell-mediated self-aggression [reviewed in Ref. (37)]. These findings strongly suggest that the interaction between NK and self-cells during infection and/or inflammation should be the rule and not the exception; in addition, NK cells together with the so-called T cells with NK activity (primarily NKT and γδT cells) can down-regulate or even impede the generation of an adaptive immune response (43, 65, 66). It is conceivable that this interaction does not happen in the peripheral blood but within tissues or in the lymph nodes, at least in the case of organ-specific autoimmune diseases. In this context, several evidences have been reported on the presence of NK, NKT, or γδT lymphocytes, expressing NKG2D and DNAM1, among tissue infiltrating cells during autoimmune diseases; in the same tissues NKG2D and/or DNAM1 ligands are detectable. Indeed, these cells have been found in psoriatic, blistering diseases, and alopecia areata (AA) skin lesions (16, 22, 67–71), central nervous system (CNS) in multiple sclerosis (MS) patients (23, 25, 72–78) and synovial fluid in rheumatoid arthritis (RA) (17, 79–83).

## Mesenchymal Stromal Cells as a Target for NK Cells

**Figure 1:**
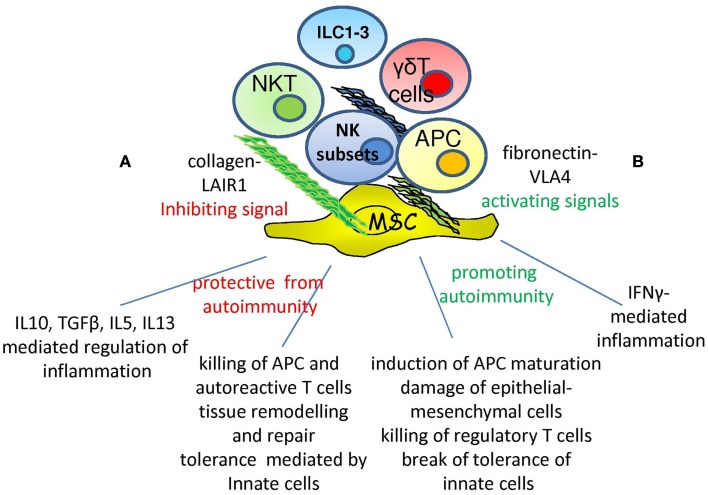
**Opposite roles of NK cells in autoimmunity**. Within microenvironment the interaction of specific NK cell receptor with extracellular-matrix can deliver different signals depending on the type of receptor involved: LAIR1–collagen interaction would lead to inhibition while VLA4-fibronectin engagement to activation. Depending on the type of NK cell subset involved, NK cells show the ability of protecting from the occurrence of autoimmunity **(A)** through the secretion of immune-regulating cytokines as IL10, TGFβ, IL5, and IL13. In addition, they can eliminate APC and autoreactive T cells through the triggering of activating receptors or regulate tissue homeostasis. On the other hand, NK cells can aggress tissues inducing inflammation through IFNγ production **(B)**, favoring the maturation of APC with the consequent triggering of adaptive immune response. Killing and damaging parenchymal, epithelial, and mesenchymal cells eventually lead to an altered tissue homeostasis and then to autoimmunity. ILC1-3, NKT, and γδT cells are involved and may regulate the NK cell–microenvironment interactions.

Within tissues NK cells can interact with other cells of innate immunity as monocyte-derived macrophages and dendritic cells, mesodermal cells, and extracellular-matrix components (EMCs) besides NKT and γδT cells (Figure 1). In particular, mesenchymal stromal cells (MSCs) are fibroblast-like cells responsible for the production of several extracellular-matrix proteins as collagen, vitronectin, fibronectin, and laminin, through which parenchymal cells can maintain both shape and functional interactions in a given organ. Among MSC, mesenchymal stem cells can undergo differentiation to stromal cells typical of connective tissues, including osteocytes, adipocytes, and chondrocytes [reviewed in Ref. (84)]. According to some experimental findings, the property to differentiate is not limited to cells of mesodermal origin but is also shared by ectodermal cells as neurons. Although conflicting results are reported in the literature, it is becoming evident that MSC can be a source of pluripotent stem cells that can be employed in tissue repair and regeneration. In addition, a functional common feature of MSC is the ability of regulating immune responses [Ref. (85); reviewed in Ref. (84, 86)]. Indeed, it has been shown that MSC derived from different tissues can down-regulate the activation of the immune system both *in vitro* and *in vivo* murine models. More importantly, these cells have been proposed as an additional therapeutic tool to control graft versus host disease (GVHD) in particular in children (87, 88). MSC can have a role in regulating autoreactivity through the modulation of cell-to-cell interactions and the production of extracellular-matrix proteins, cytokines, and enzymes [Ref. (85); reviewed in Ref. (84)]. The prevailing point of view of the literature is that MSC have a regulatory inhibiting role on several T and NK cell-mediated activities (87, 89). This regulation is reported to be mediated by soluble factors, such as TGFβ, HGF, IDO, and PGE2, which affect lymphocyte functions upon lymphocyte–MSC interaction [Ref. (85); reviewed in Ref. (84, 86, 89)]. On the other hand, it appears that NK and T cells can aggress MSC recognizing NKG2D and DNAM1 ligands, leading to MSC killing and release of pro-inflammatory cytokines (57, 90–92). This property is mainly confined to cytokine-activated NK cells, as *ex vivo* peripheral blood isolated NK cells are not efficient in MSC killing (90–93). It is of note that the regulatory role of MSC on NK cell functions is found *in vitro* at well defined MSC:NK cell ratios, ranging from 1:1 to 1:4, while at lower MSC–NK cell ratios the inhibiting effect is barely or not detectable and an activating effect is found (90). Due to *in vitro* culture conditions, at this ratios MSC grow as a monolayer covering the culture well, with lymphocytes seeded on them; thus, both extracellular-matrix proteins, as collagen and fibronectin, and inhibiting cytokines, as TGFβ, can be concentrated to the MSC surface and in the extracellular medium facilitating the delivery of an inhibiting signal to lymphocytes. On the other hand, it is still to be determined what happens during the interaction of a single MSC and an NK cell: it is conceivable that within connective tissues MSC–NK interactions take place in the presence of several extracellular-matrix proteins whose receptors are expressed on NK cells.

## Extracellular-Matrix Protein Receptors on NK Cells as Regulators of NK Cell Functions: Focus on the Leukocyte Associated Ig-Like Receptor 1

Natural killer cells can express different extracellular-matrix protein receptors as well as matrix metalloproteinases responsible for matrix degradation (94, 95). Some of these receptors are called very late antigen (VLA) as they are expressed on long-term cultured cells (Figure 1). However, some are constitutively expressed at the NK cell surface, such as VLA4 (96–98), and can also be up-regulated upon stimulation. Several different effects of NK cell interaction with the matrix proteins fibronectin, laminin, vitronectin, osteopontin, and collagen are reported in the literature (99–105) and their deep analysis is beyond the scope of this review. As an example, the engagement of VLA4 can induce activation of NK cells (98, 106–109). Herein, we focus on the leukocyte associated Ig-like receptor 1, LAIR1 or CD305 (110, 111), that has been shown to be a receptor for the Gly-Prol-Hyp common motif of collagens type I, II, III, XIII, XVII, and XXIII (112–115) (Figure 1). Importantly, LAIR1 is able to deliver an inhibiting signal which down-regulates NK cell activation through the CD16 receptor, reducing calcium mobilization, and the cytolytic activity triggered through this molecule (110, 116, 117). The LAIR1-mediated inhibiting signal occurs through the recruitment, by its cytoplasmic tail equipped with immunoreceptor tyrosine inhibiting motif (ITIM), of the SHIP1 phosphatase; this, in turn, impedes the phosphorylation and consequent activation of the immunoreceptor tyrosine activating motif (ITAM) present in the intracellular domain of several activating NK cell receptors (2, 118). LAIR1 can be expressed as different isoforms (LAIR1a, b, and c) or as a soluble form termed LAIR2; it is conceivable that the interaction of NK cells with collagens delivers a negative signal that may be impaired in the presence of soluble (s) LAIR (119–121). No direct evidence for the interaction of LAIR1 expressed by NK cells and collagen is reported so far; however, that indeed cross-linking of collagen can trigger an inhibiting signal in lymphocytes upon LAIR1 engagement has been demonstrated for T and B cells (117, 122–124), APCs (125, 126), and tumor cells (127–130). Altogether, these findings suggest that collagen produced by MSCs may be involved in the negative regulation of NK cell function. It is still to be defined which stimuli can regulate LAIR1 expression on NK cells. It is of note that LAIR1 is present on almost all leukocytes and it appears to be associated with the leukocyte common antigen (LCA) tyrosine phosphatase (CD45) on NK cells (131); thus, LAIR1 could regulate NK cell activation by itself and/or through the association with CD45. Interestingly, the lack or lower expression of LAIR1 is associated with an impaired inhibiting signal delivered upon LAIR1 engagement in B cells isolated from systemic lupus erythematosus (SLE) patients or B cell chronic leukemia (129, 132) supporting the idea that down-regulation of LAIR1 expression can be associated with autoimmune or neoplastic diseases.

## NK Cell Subsets and Innate Lymphoid Cells as Players and Regulators of Autoimmunity

It is generally thought that autoreactivity and autoimmune diseases are based on an altered adaptive immune response determining the generation of T and B cell-mediated aggression of self-cells (133–136). This can be the result of a too strong reaction to self-antigen due to altered central or peripheral tolerance of autoreactive T and B cell clones. Treg cells are the main effectors of tolerance and several evidences have demonstrated that the lack of an optimal regulation of the adaptive immune response may be a consequence of their impaired function (137). NK cells can influence tolerance by eliminating Treg cells (15, 138, 139) or by acting as regulatory cells themselves (14, 21, 140–144). Indeed, upon engagement of activating receptors, NK cells can release several regulating cytokines, such as TGFβ and IL10, which are considered mediators of tolerance for T cells (5, 145). For instance, during viral infections, it is conceivable that the interaction of NK cells with infected self-cells results in the secretion of TGFβ and IL10, which in turn modulate T and B cell responses; of note, TGFβ is a strong down-regulator of NK cell-mediated activation and proliferation (146–149). Interestingly, secretion of functional TGFβ can be elicited in NK cells upon triggering with soluble HLA-I molecules that interact with the corresponding counter-receptors, as CD8 and/or the activating isoforms of KIRs and/or CLIRs (150, 151). An increment of sHLA-I can be detected in the sera of patients suffering from different autoimmune diseases; thus, one could suggest that sHLA-I can down-regulate NK cell activation. In addition, together with TGFβ, NK cells can release FasL (152); in turn, soluble FasL, interacting with Fas at the surface of lymphocytes, can lead to their cell death. Thus, the NK cell-mediated down-regulation of immune response may occur both by blocking activation with TGFβ and triggering cell death via FasL–Fas interaction (152). Recently, several distinct NK cell subsets have been found in different tissues playing opposite functional roles in immune response (Table 1). Briefly, it is commonly accepted that CD56^dull^ and CD56^bright^ NK cells present in the peripheral blood have distinct phenotype and functional activities. Indeed, CD16^+^KIR^+^CD56^dull^ NK cells are primarily cytotoxic while the CD16^−^KIR^dull^ CD56^bright^ produce huge amounts of cytokines. It is not clear whether CD56^dull^ posses the plasticity to become CD56^bright^ and viceversa. Also, human NK cells can be subdivided on the basis of CD27 and CD11b expression (153, 154): the minority of peripheral NK cells is CD27^+^ (about 5%), while this population is more represented in the bone marrow and further in the spleen and tonsils. CD27^+^ NK cells, either CD11b^+^ or CD11b^−^, can produce high amounts of cytokines while among the CD27^−^ NK cells those expressing CD11b are highly cytotoxic (Table 1). Of note, early during pregnancy the majority of human decidual lymphocytes are characterized by unique phenotype: CD16^−^CD11b^−^CD56^bright^ either expressing or not CD27, CD9, and CD151 tetraspanning family members. Some of these cells can produce IL22 and express immunomodulatory molecules as galectin-1 and progestagen-associated protein 14 (155). Importantly, decidual NK (dNK) CD56^bright^CD27^+^ cells suppress Th17 through an IFNγ-dependent pathway and this population is lost in women with spontaneous abortion. Additional NK cell subsets, as NK2, NK3, NKr, and NK22 specifically involved in the secretion of immune-regulatory cytokines have been recently identified [reviewed in Ref. (21, 156–160)]. Subsets with a protective role in autoimmunity are NK2 cells, predominant in allergic disease, producing high amounts of IL4, IL5, and IL13 (161), NK3 cells which release IL10 (162), together with secreting TGFβ NKr cells which are involved in maternal-fetal immune tolerance (80) while NK22 cells limit inflammation and protect gut mucosal integrity through the action of IL22. To further complicate this scenario, innate lymphoid cells (ILCs, Table 2) distinct from NK cells, has been identified in mucosa associated lymphoid tissue (163). To uniform this variegate picture, it has been suggested to include NK cells within the ILC1 subset and it has been proposed that the CD56 molecule can be considered the best marker to distinguishing between NK and other lineage negative lymphoid cells (Table 2) as both kind of cells can express NKp46 and NKp44 receptors. More importantly, ILC1, ILC2, and ILC3 subsets express peculiar transcription factors as T-bet or RORα or RORγT (Table 2) resembling Th1, Th2, or Th17 T cell subsets respectively. Of note, ILC1, ILC2, and ILC3 cells are present in the gut and display a pro-inflammatory or a protective role depending on the main cytokine produced (Table 2). Finally, the NKp46^−^NKp44^+^RORγT^+^CD127^+^ NK cells show a protective role in autoimmunity but they may be counteracted by NKp46^+^NKp44^−^RORγT^−^CD127^−^ NK cells which appear to be pathogenic through the production of IFNγ (164). Altogether these findings suggest that both different NK cell subsets and ILC are primarily involved in either host defense against viruses and tumor immunosurveillance or in regulating tissue homeostasis and autoimmunity. Furthermore, it is still to be determined the “plasticity” of an NK cell or ILC subset as it has been demonstrated for some T cell subsets [reviewed in Ref. (165, 166)].

**Table 1:** **Features of NK cell subsets in peripheral blood and tissues**.

NK cell type	Cytotoxic	Regulatory/tolerant
Prototype examples	Peripheral NK cells	Decidual NK cells
		Liver NK cells
		Tissue infiltrating NK cells
Phenotype	CD56^dim^CD27^−^CD11b^+^	CD56^bright^CD27^−^CD11b^−^
		CD56^brigth^ or CD27^+^
Cytokine produced	Mainly IFNγ and TNFα	Several different cytokines (TGFβ, VEGF, IL10, IL17, IL22)
Main activity	Cytolysis	Vascular remodeling
		Maternal-fetal immune regulation
Tissue localization	Peripheral blood, bone marrow	Lung, uterus, liver, and gut
Immunity against	Viruses and tumor immunosurveillance	Maintenance of tissue homeostasis
Role in autoreactivity	Triggering or protective effect	Mainly protective effect

*Schematically, NK cells can show two different functional behaviors (a) cytolytic NK cells (cNK) express high levels of lytic granules and kill spontaneously tumor cells; (b) regulatory/tolerant NK cells producing several soluble factors which are relevant in regulating tissue homeostasis. Cytotoxic NK cells may exert a key role in inducing inflammation and they can down-regulate adaptive immunity acting on antigen presenting cells. Regulatory/tolerant NK cells are involved in controlling tissue homeostasis playing a protective role aimed to maintain and reconstitute the healthy conditions during tissue reparation*.

**Table 2:** **Innate lymphoid cells characteristics**.

Characteristic	ILC group 1	ILC group 2	ILC group 3
Cell type	NK cells (CD56^dim/bright^ NKp46^+^)	IL1R^+^	ILC3 and LTi cells
	ILC1 cells not cytotoxic	IL23R^+^	Some CD56^+^ cells
Main cytokine produced	IFNγ	IL5 and IL13 triggered through IL25 or IL33	IL22 and/or IL17
			IFNγ
Cytolytic activity	Yes (NK cells)		
Main transcription factor expressed	T-bet	RORα	RORγt
	Eomes	GATA3	
Peculiar phenotypic features	c-kit^−^ (CD117)	c-kit^−^	c-kit^+^
	IL12Rβ2^+^	IL12Rβ2^−^	IL12Rβ2^−^
		Subunits of IL25R and IL33R	
Common phenotypic features	IL7Rα^+^ (CD127)	IL7Rα^+^	IL7Rα^+^
	NKRP1a^+^ (CD161)	NKPRP1a^+^	NKRP1a^+^
Immune function	Viral infections, tumor surveillance	Tissue defense/homeostasis	Inflammation (IL17^+^IFNγ^+^ colitis)
	NK IFNγ Inflammation (ILC1)		Protection (*Citrobacter rodendrium* ILC3 IL22^+^)
			Gut barrier, wound healing, and epithelial proliferation
			Lymph node formation (IL17^+^)
Main tissue localization	Lymphoid organs, uterus, lung, gut, liver	Lung, adipose tissue, gut	Gut lamina propria and cryptopatches, mesenteric lymph nodes, palatin tonsil
Role in autoreactivity	IBD (CD56^bright^ NK cells)	Murine models of gut parasitic infections	Murine models of colitis
		Human IBD	Chron disease

*Innate lymphoid cells (ILCs) are mucosa associated lymphoid cells which can express some markers of NK cells. It has been proposed to include NK cells within the ILC1 subset of ILC. NK cells are CD56^+^ and display strong cytolytic activity while ILC produce a set of different cytokines depending on the subset they belong to. This dichotomy is not so well defined as some ILC3 cells can express CD56 and thus it is still debate whether NK and some ILC subsets may be inter-converted due to intrinsic plasticity. IBD, inflammatory bowel disease*.

## NK Cells and Genetic Correlation with Autoimmune Diseases

**Figure 2:**
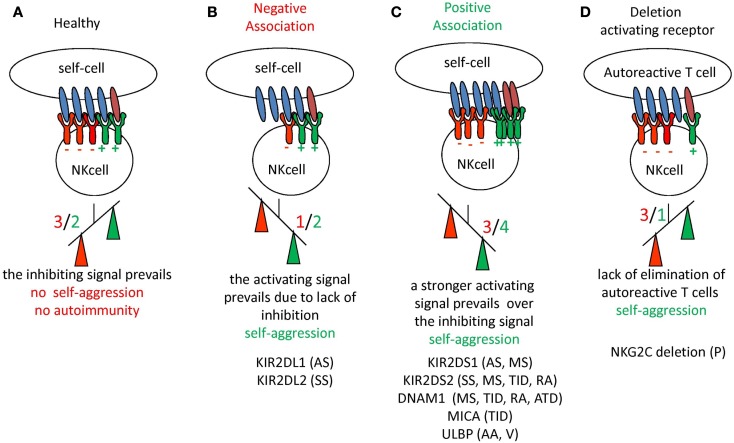
**Schematic representation of the imbalance between inhibiting and activating receptors on NK cells with the occurrence of autoimmunity and autoimmune disease**. **(A)** In healthy conditions the negative signal (in red) delivered through inhibiting receptors for HLA-I can prevail on the positive signal (in green) induced by the engagement of activating receptor. The net effect is that no damage to self-cells is induced, thus there is no autoreactivity. **(B,C)** The down-regulation of expression of inhibiting receptors **(B)** or an increment of activating receptors **(C)** determines in any case the prevalence of the activating signal on the inhibiting one, leading to self-reactivity (indicated as prevalence of positive signal shown in green). Although not shown, these two situations may also be found together. **(D)** In this case the lack of activating receptors can lead to impaired blocking of autoreactive T cell clones favoring autoreactivity. For each situation are listed the autoimmune diseases where an alteration of NK receptors have been reported. KIR2DS1 and KIR2DS2 are the activating isoforms of the NK receptor for HLA-C alleles. The KIR2DL1 and KIR2DL2 are the inhibiting isoforms of NK receptors for HLA-C alleles. NKG2C is the activating isoform of CLIR. DNAM1 is an activating receptor present on NK cells and MICA and ULBP are the ligands of the NKG2D surface receptor involved in the recognition of either infected or tumor transformed cells. AA, Alopecia areata; AS, ankylosing spondylitis; ATD, autoimmune thyroid disease; MS, multiple sclerosis; P, psoriasis; PV, pemphigus vulgaris; RA, rheumatoid arthritis; SS, systemic sclerosis; TID, type I diabetes; V, vasculitis.

Natural killer cell development and function is strictly related to genetic elements: the genetic background, particularly the defects and variations of KIR/HLA genotypes, can influence the function of a given NK cell receptor in target cell recognition and impair NK cell activation as well as self-tolerance. This influence is supposed to be related to autoimmunity (167); indeed, several findings have pointed out associations between risk of systemic or organ-specific autoimmune diseases and KIR/HLA genotypes, which indicate that self-tolerance may be broken with inappropriate receptor and ligand pairs or with the interrupted signal balance (38, 168–177). In general, the presence of an activating receptor for HLA-I associated with the lack or reduction of inhibitory pairs has been shown in several autoimmune diseases [reviewed in Ref. (21, 178)] suggesting that an imbalance in favor of activating receptors for HLA-I is associated with autoimmunity (Figure 2). This is in line with the findings observed in bone marrow transplantation where the expression of activating KIRs can override the regulating signals generated through inhibiting KIRs and/or CLIRs (179–182).

In the following paragraphs, the findings regarding the potential role of NK cells in different autoimmune diseases will be listed and discussed.

## Features of NK Cells in Skin Diseases

Typical autoimmune diseases of the skin are psoriasis, pemphigus vulgaris (PV), and AA; herein, we will not deal with skin specific manifestations of SLE which can be considered as a systemic autoimmune disorder with involvement of the skin. It has been reported that NK cells represent about 5–8% of infiltrating lymphocyte in psoriatic lesions and these cells are CD56^bright^CXCR3^+^CCR5^+^ cells (67) expressing the activation antigen CD69. These cells produce IFNγ after IL2 stimulation; in turn IFNγ can upregulate the HLA-I antigens on cheratinocytes and trigger activation of these cells as well. These findings would suggest that CD56^+^ NK cells can favor the development of psoriasis inducing local inflammation and amplify T cell autoimmune reactivity. This notion is further supported by the finding that CD56^bright^CXCR3^+^CCR5^+^ NK cells from psoriatic lesions can trigger cheratinocytes to produce CCL5 and CXCL10 chemokines which in turn favor NK cell chemotaxis. Of note, NK cells can also release IL22, a cytokine mainly produced by Th1 and Th17 T cells (183–185). Cheratinocytes incubated with IL22 can proliferate upon interaction with IL22 receptor (186) and this leads to parakeratosis and acanthosis, typical features of psoriatic lesions (187). Furthermore, genomic deletion of the activating receptor NKG2C is significantly increased in psoriatic patients compared to healthy matched controls (70, 188, 189). Thus, the lack of recognition by NK cells of autoreactive T cells may lead to exacerbation of psoriasis (Figure 2). In this context, the finding that NK cells bearing the inhibiting NK receptor NKG2A are incremented in skin psoriatic lesions can suggest that the imbalance between NKG2C^+^ and NKG2A^+^ NK cells may favor the expansion of autoreactive T cells (70). In AA, it has been found that CD56^+^NKG2D^+^ NK cells are mainly localized around and within the anagen hair follicles in prominent aggregates possibly leading to aggression of hair follicles favoring the collapse of the relative immune privilege of this cutaneous region (69). Finally, in PV it has been reported that peripheral NK cells display a Th2 type-biased phenotype (190) as they express high mRNA for IL10, a decrement of IL12Rβ, and produce IL5 *in vivo*, exclusively in patients with active disease compared to healthy control. Furthermore, NK cells may function as APCs for desmoglein three antigens to CD4^+^ T cells, suggesting also the possibility of a role for NK cells in inducing the tissue damage associated to PV (191).

## NK Cells in Multiple Sclerosis

Multiple sclerosis is a CNS inflammatory autoimmune disease involving as target the myelin associated with neuronal axons; MS eventually leads to a progressive disability and host death due to the impairment of vital CNS functions. A potential pathogenic role of NK cells in MS is supported mainly in relapsing remitting MS patients (RRMS) [reviewed by Chanvillard et al. (23)]; indeed, NK cells can directly aggress and damage oligodendrocytes which produce myelin and NK cells are increased in MS lesions (192, 193). On the other hand, NK cells can directly affect the life of autoreactive T cells or APCs; in MS patients treated with IFNβ (194) or with the anti-CD25 antibody daclizumab, there is a selective expansion and activation of CD56^bright^ NK cells and this correlates with a down-regulation of T cells activation and inhibition of inflammation (195, 196), suggesting that CD56^bright^ NK cells are relevant in the control of MS lesions. Importantly, this NK cell subset appeared to kill T cells through granzyme K and A, which activate the mitochondrial pathway of apoptosis. The expansion of CD56^bright^ NK cells can be dependent on their relative higher expression (compared to CD56^dull^ NK cells) of the intermediate affinity receptor for IL2. Thus, during MS therapy CD56^dull^ NK cells should be shut down through the blocking of the CD25 receptor by daclizumab; on the other hand, the expansion of CD56^bright^ NK cells is favored because daclizumab does not impair their proliferation. Furthermore, in MS the NK2 cell subset is responsible for the production of IL5 and IL13, which may actively suppress the activity of self-reactive T cells. These cells disappear in MS patients when an exacerbation of the disease is present, while they re-appeared during the remission phase, suggesting that NK2 cells may be relevant for the disease control (197, 198).

## NK Cells and Type I Diabetes

A reduction of peripheral NK cells has been reported in early diagnosed type I diabetes (TID) while the amount of NK cells is mostly similar to healthy controls in long-standing TID patients; more importantly, long-standing TID display lower amounts of IFNγ and lower expression of some natural cytotoxicity receptors (199) associated with high levels of glycosylated hemoglobin, suggesting that the impairment of NK cells could be a consequence of the disease. It is of note that some NK cells have been identified also within the pancreas, nearby to β pancreatic islets (200), although this finding has not been confirmed (201). In a murine diabetes model, it has been reported that NK cell are essential in abolishing the onset of the disease in NOD mice through a TGFβ-dependent mechanism that interferes with the activation of β-islet specific T cells (202, 203).

## NK Cells in Rheumatoid Arthritis

It has been reported that NK cells producing IL22 and TNFα are increased in the synovial fluid of RA patients. It is of note that culture supernatants from these IL22-producing NK cells can trigger the proliferation of synovial fibroblast-like synoviocytes and this proliferation is inhibited using anti-IL22 and anti-TNFα antibodies (79). In addition, NK cells from synovial fluid are mainly CD56^bright^, express high levels of activation antigens and produce IFNγ. Furthermore, they can induce monocyte differentiation to dendritic cells, which in turn can trigger NK cells (204). Altogether these findings would suggest an active role of NK cells in sustaining inflammation in RA patients.

## NK Cells in Inflammatory Bowel Disease

Inflammatory bowel diseases are represented by ulcerative colitis (UC) and Chron disease (CD): these two illnesses are characterized by the inflammation of gut accompanied by diarrhea and impairment of absorption of nutrients. It is commonly accepted that IL17A-producing lymphocytes are extremely relevant in IBD (205); among the different cell populations residing and colonizing (Th17, Th1-Th17, NKT, γδT cells) bowel mucosa in UC or CD, NK cells, and the group 3 of ILC3 (see Table 2) are good producer of IL17A. These cells can release IL17A immediately upon engagement with pathogen associated molecular patterns (PAMPs) and/or cytokines as IL23 (163). More importantly, both NK cells and ILC3 producing IFNγ and IL17 are abundant in inflamed CD mucosa while it is debated whether ILC3 secreting IL22 cells are increased or decreased in IBD (164, 206). It appears that some ILC of the subgroup 3 (Table 2) are relevant in the generation of the gut-associated lymphoid tissues and the maintenance of healthy conditions. In this context, the fine tuning of the respective functional role of colitogenic ILC producing IFNγ (ILC1 and some ILC3) and protective ILC3 secreting IL22 should be relevant in the generation of IBD.

## NK Cells in Autoimmune Liver Diseases

Natural killer cells present in the healthy liver are different from those found in the peripheral blood; indeed, the former are mainly CD56^dull^ and about a half do not express CD16. Furthermore, these cells are more prompt to be stimulated with IL2 and, unexpectedly, do not lyse autologous hepatocytes, although these cells do not bear HLA class I antigens [reviewed in Ref. (207)]. Autoimmune diseases that hit the liver are mainly represented by AIH, primary biliary cirrhosis (PBC), and primary sclerosing cholangitis (PSC). AIH is characterized by the progressive destruction of the liver parenchyma which eventually leads to cirrhosis and in several instances to hepatic failure and host death. NK cells, together with γδT cells, play a role in the physiopathology of the AIH (208, 209). This is confirmed also in a murine model where administration of poly immune complexes (IC) can induce a strong production of type I IFN and consequent activation of liver NK cells leading to liver destruction with similar histopathologic features found in human AIH (210). In PBC, besides IL17^+^ cells infiltrating damaged bile ducts, hepatic NK cells active against biliary epithelial cells are found, but it is to be determined whether they are directly involved in the break of immune tolerance characteristic of this disease (207, 211–213). PSC is characterized on one hand by the reduced frequencies of some alleles of inhibiting receptors for HLA-I (214) and on the other by the expression of peculiar alleles of the NKG2D ligand MICA (215); both these molecular events might regulate the NK cell-mediated immune interaction with cholangiocytes.

## NK Cells in Lupus Erythematosus Systemicus

Systemic lupus erythematosus is a systemic autoimmune disease characterized by tissue damage mediated mainly through type II and III hypersensitivity. Several autoantibodies are present in SLE patients and it is evident that interaction with cellular antigens can deliver an activating signal to leukocytes bearing Fcγ receptors, as NK cells and monocyte–macrophages, which eventually leads to cell damage and inflammatory cytokine production. In SLE, a reduction of the absolute number of NK cells with an impaired cytolytic activity is reported (20, 216–222) with an imbalance between CD56^dull^ and CD56^bright^ peripheral blood NK cell subsets (223) characterized by an increase of cytokines production (220) and a lower lymphokine activated killer cell activity (219). It is of note that in different systemic autoimmune disorders, as systemic sclerosis (SSc) and anti-neutrophil cytoplasmic antibody-associated vasculitis, the number of CD3^−^CD56^+^ NK cells are markedly reduced (224). These findings could be interpreted either as a consequence or as a pathogenic player of the autoimmune disorder. In addition, the NK cell subsets found in the peripheral blood may be considered as the results of the localization of effector cells within target tissues, mainly in the case of systemic autoimmune diseases (24, 76). Recently, it has been reported a prominent reduction of NK cells expressing the DNAM1 activating receptor together with an up-regulation of DNAM1 ligand on plasmocytoid dendritic cells (pDCs) which in turn can mediate NK cell death through type I IFNα (20). Of note, in the MRL-lpr/lpr mice model kidney-infiltrating NK cells express activation antigens and high content of cytotoxic granules, suggesting a possible role in the kidney tissue damage associated with SLE (20). The presence of autoantibodies to inhibitory NK cell receptors and NKG2A (225) can promote excessive NK cell function leading to increased levels of autoantigens and further stimulating autoimmune reactions. Of note, in SLE an increase of CS1/CD319 activating receptor of the SLAM family on NK and pDCs could be detected upon triggering with RNA-IC (225); in addition, expression of CS1/CD319 on B cells of SLE patients increased. Altogether, these findings would suggest a role of CS1/CD319 homophylic interaction among pDC, NK, and B cells in SLE (226, 227). It is still to be determined whether these interactions are involved in the pathogenesis of SLE and whether NK cells may be protective or not in this disease.

## Authors’ Viewpoint

**Figure 3:**
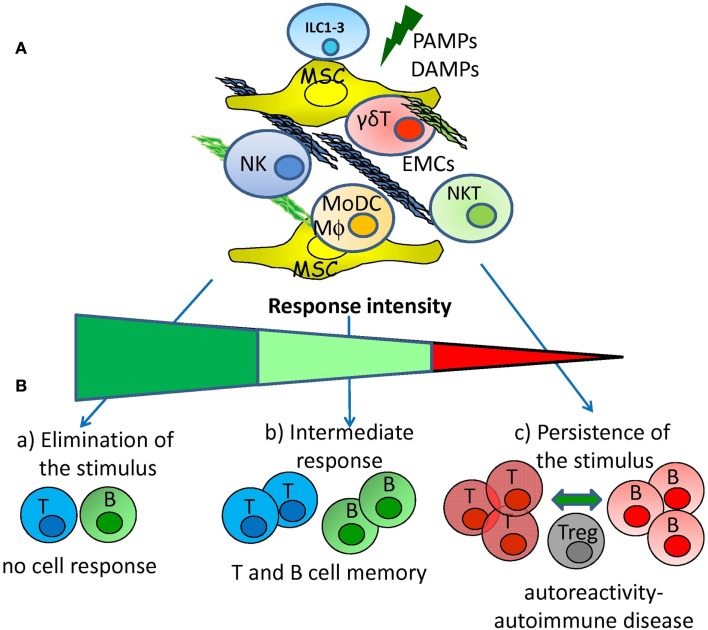
**Hypothesis for the generation of adaptive autoreactivity and autoimmunity**. **(A)** Pathogen associated molecular patterns and/or damage associated molecular patterns (PAMPs and DAMPs) can activate innate immunity interacting with receptors expressed on innate lymphoid cells (NK, ILC subsets, NKT, and γδT lymphocytes). The activation of innate immunity can be regulated by reciprocal interactions among mesenchymal stromal cells (MSC), extracellular-matrix components (EMCs), lymphoid cells, monocyte-derived macrophages (MΦ), and dendritic cells (MoDCs). **(B)** Innate response elicited by NK, ILC subsets, NKT, and γδT lymphocytes interacting with MSC and EMCs can lead to: (a) rapid elimination of the danger signal that avoids the triggering of adaptive immune cell response; (b) intermediate innate response that leads to the triggering of adaptive immunity with the generation of memory T and B cells; (c) low innate response that determines the persistence of the danger signal leading to generation of autoreactive T and B cells. Autoreactive T and B lymphocytes are controlled by regulatory cells (Treg) but chronic stimulation tends to break the tolerance leading to autoimmune disease.

It is clear that antigen unspecific autoreactivity can occur, before the onset of an autoimmune disease or in healthy individuals that will not develop any illness: cytotoxic NK (some ILC1 cells), NKT, and γδT cells, are the main active players of this phenomenon while regulatory/tolerant NK cells and ILC2 and ILC3 are mainly involved in maintaining tissue homeostasis. The complex cellular network composed of effector lymphocytes, MSCs, and APCs is the place where the fate of antigen unspecific reactivity determines whether adaptive immune responses will take place or not. One could hypothesize that a strong innate immunity can impede the generation of adaptive immunity as infectious agents are cleared before specific T and B lymphocyte can respond. On the other hand, a low innate response chronically triggers specific T and B cells favoring the establishment of an autoimmune disease due to persistence of the antigen. Finally, an adequate innate response can lead to an optimal B and T cell response that definitively clear the antigen without self-aggression as a consequence (Figure 3). If this idea is true, to cure an autoimmune disease one should trigger innate immunity instead of down-regulate adaptive immunity. However, any therapeutic treatment should take into account that both innate and adaptive immune responses can be regulated through MSCs and EMCs besides lymphocytes and APC.

## Author Contributions

Both the authors have equally contributed to the preparation and reviewing of this manuscript.

## Conflict of Interest Statement

The authors declare that the research was conducted in the absence of any commercial or financial relationships that could be construed as a potential conflict of interest.
